# Supplementary material for: Nanoscaled RIM clustering at presynaptic active zones revealed by endogenous tagging
Source: Life Sci Alliance. 2023 Sep 11;6(12):e202302021. doi: 10.26508/lsa.202302021 (PMC10494931; doi:10.26508/lsa.202302021)
Supplement: Supplementary file 1 [file LSA-2023-02021_TableS1.docx]

| **parameter** | **wt (w^1118^)** | **rim^rescue-Znf^** | **rim^V5-Znf^** | **rim^HA-Znf^** |
| --- | --- | --- | --- | --- |
| **mEPSC amplitude**  **[-nA]** | 0.707 ± 0.032  n = 15 NMJs,  7 larvae | 0.663 ± 0.016  n = 17 NMJs,  8 larvae | 0.658 ± 0.030  n = 12 NMJs,  6 larvae | 0.696 ± 0.035  n = 12 NMJs,  5 larvae |
| **mEPSC frequency**  **[Hz]** | 2.038 ± 0.268  n = 15 NMJs,  7 larvae | 1.741 ± 0.175  n = 17 NMJs,  8 larvae | 2.469 ± 0.310  n = 12 NMJs,  6 larvae | 2.075 ± 0.197  n = 12 NMJs,  5 larvae |
| **mEPSC rise time**  **[ms]** | 1,293 ± 0.051  n = 15 NMJs,  7 larvae | 1.222 ± 0.039  n = 17 NMJs,  8 larvae | 1.285 ± 0.074  n = 12 NMJs,  6 larvae | 1.246 ± 0.035  n = 12 NMJs,  5 larvae |
| **mEPSC tau decay**  **[ms]** | 9.376 ± 0.207  n = 15 NMJs,  7 larvae | 8.965 ± 0.243  n = 17 NMJs,  8 larvae | 9.314 ± 0.191  n = 12 NMJs,  6 larvae | 9.507 ± 0.107  n = 12 NMJs,  5 larvae |
| **eEPSC amplitude**  **[-nA]** | 40.99 ± 2.67  n = 10 NMJs,  7 larvae | 38.45 ± 2.39  n = 13 NMJs,  7 larvae | 27.41 ± 1.96  n = 12 NMJs,  5 larvae | 37.53 ± 2.30  n = 11 NMJs,  5 larvae |
| **eEPSC rise time**  **[ms]** | 1.054 ± 0.023  n = 10 NMJs,  7 larvae | 1.022 ± 0.026  n = 13 NMJs,  7 larvae | 1.054 ± 0.014  n = 12 NMJs,  5 larvae | 1.017 ± 0.020  n = 11 NMJs,  5 larvae |
| **eEPSC tau decay**  **[ms]** | 9.066 ± 0.131  n = 10 NMJs,  7 larvae | 8.774 ± 0.282  n = 13 NMJs,  7 larvae | 9.126 ± 0.145  n = 12 NMJs,  5 larvae | 9.052 ± 0.242  n = 11 NMJs,  5 larvae |
| **PPR**  **(30 ms IPI)** | 1.171 ± 0.017  n = 12 NMJs,  6 larvae | 1.160 ± 0.028  n = 11 NMJs,  6 larvae | 1.243 ± 0.041  n = 12 NMJs,  5 larvae | 1.217 ± 0.017  n = 10 NMJs,  5 larvae |

**Table S1. Electrophysiological analysis of spontaneous and evoked synaptic transmission in rim^rescue-Znf^, rim^V5-Znf^ and rim^HA-Znf^. Related to Figure 2 A-D.** Numerical values are presented as mean ± SEM for each genotype. Sample sizes for the number of NMJs and the number of animals used for analysis are indicated. For statistical comparison see Table S2.
