# Supplementary material for: Nanoscaled RIM clustering at presynaptic active zones revealed by endogenous tagging
Source: Life Sci Alliance. 2023 Sep 11;6(12):e202302021. doi: 10.26508/lsa.202302021 (PMC10494931; doi:10.26508/lsa.202302021)
Supplement: Supplementary file 2 [file LSA-2023-02021_TableS2.docx]

| **genotype** | **mEPSC amplitude** | **mEPSC frequency** | **mEPSC rise time** | **mEPSC tau decay** | **eEPSC amplitude** | **eEPSC rise time** | **eEPSC tau decay** | **PPR**  **(30 ms ISI)** |
| --- | --- | --- | --- | --- | --- | --- | --- | --- |
| wt (w^1118^)  vs. rim^rescue-Znf^  vs. rim^V5-Znf^  vs. rim^HA-Znf^ | 0.635  0.614  0.994 | > 0.999  > 0.999  > 0.999 | > 0.999  > 0.999  > 0.999 | 0.447  0.997  0.972 | > 0.999  0.005  > 0.999 | > 0.999  > 0.999  0.652 | 0.786  0.998  > 0.999 | > 0.999  0.524  > 0.999 |
| rim^rescue-Znf^  vs. rim^V5-Znf^  vs. rim^HA-Znf^ | 0.999  0.831 | 0.250  > 0.999 | > 0.999  > 0.999 | 0.634  0.261 | 0.003  > 0.999 | > 0.999  > 0.999 | 0.640  0.797 | 0.150  0.395 |

**Table S2. Statistical comparison of spontaneous and evoked synaptic transmission in rim^rescue-Znf^, rim^V5-Znf^ and rim^HA-Znf^. Related to Figure 2 A-D.** p-values revealed by one-way ANOVA for parametric data (mEPSC amplitude, mEPSC tau decay, eEPSC tau decay) or by Kruskal-Wallis test for non-parametric data (mEPSC frequency, mEPSC rise time, eEPSC amplitude, eEPSC rise time, PPR) are presented for comparisons between wt (w^1118^) and rim^rescue-Znf^, wt and rim^V5-Znf^ or wt and rim^HA-Znf^, respectively, or for comparisons between rim^rescue-Znf^ and rim^V5-Znf^ or rim^rescue-Znf^ and rim^HA-Znf^, respectively.
