# Supplementary material for: Nanoscaled RIM clustering at presynaptic active zones revealed by endogenous tagging
Source: Life Sci Alliance. 2023 Sep 11;6(12):e202302021. doi: 10.26508/lsa.202302021 (PMC10494931; doi:10.26508/lsa.202302021)
Supplement: Supplementary file 3 [file LSA-2023-02021_TableS3.docx]

| **parameter** | **rim^rescue-Znf^**  **(ctrl)** | **rim^rescue-Znf^**  **(phtx)** | **rim^HA-Znf^**  **(ctrl)** | **rim^HA-Znf^**  **(phtx)** |
| --- | --- | --- | --- | --- |
| **eEPSC amplitude**  **[-nA]** | 42.81 ± 1.89  n = 14 NMJs, 7 larvae | 43.36 ± 1.97  n = 13 NMJs, 6 larvae | 48.29 ± 3.93  n = 11 NMJs, 5 larvae | 44.34 ± 1.68  n = 13 NMJs, 5 larvae |
| **mEPSC amplitude**  **[-nA]** | 0.687 ± 0.020  n = 14 NMJs, 7 larvae | 0.339 ± 0.014  n = 13 NMJs, 6 larvae | 0.778 ± 0.055  n = 11 NMJs, 5 larvae | 0.353 ± 0.019,  n = 13 NMJs, 5 larvae |
| **quantal content** | 84.64 ± 5.36  n = 14 NMJs, 7 larvae | 174.10 ± 10.88  n = 13 NMJs, 6 larvae | 87.42 ± 10.16  n = 11 NMJs, 5 larvae | 174.00 ± 12.50, n = 13 NMJs, 5 larvae |

**Table S3. Electrophysiological analysis of acute presynaptic homeostasis in rim^rescue-Znf^ and rim^HA-Znf^ animals. Related to Figure 2 E.** Numerical values are reported as mean ± SEM for each group. Sample sizes for the number of NMJs and the number of animals used for analysis are indicated. For statistical comparison see Table S4.
