# Supplementary material for: Nanoscaled RIM clustering at presynaptic active zones revealed by endogenous tagging
Source: Life Sci Alliance. 2023 Sep 11;6(12):e202302021. doi: 10.26508/lsa.202302021 (PMC10494931; doi:10.26508/lsa.202302021)
Supplement: Supplementary file 4 [file LSA-2023-02021_TableS4.docx]

| **genotype** | **eEPSC amplitude** | **mEPSC amplitude** | **quantal content** |
| --- | --- | --- | --- |
| rim^rescue-Znf^ (ctrl)  vs. rim^rescue-Znf^ (phtx)  vs. rim^HA-Znf^ (ctrl) | 0.830  0.223 | < 0.001  0.292 | < 0.001  0.800 |
| rim^rescue-Znf^ (phtx)  vs. rim^HA-Znf^ (phtx) | 0.545 | 0.448 | 0.997 |
| rim^HA-Znf^ (ctrl)  vs. rim^HA-Znf^ (phtx) | 0.331 | < 0.001 | < 0.001 |

**Table S4. Statistical comparison of acute presynaptic homeostasis in rim^rescue-Znf^ and rim^HA-Znf^. Related to Figure 2 E.** p-values revealed by parametric t-test (quantal content) or by Mann-Whitney Rank Sum tests for non-parametric data (eEPSC and mEPSC amplitude) are reported for comparisons between both genotypes or between measurements in DMSO (ctrl) and PhTx (phtx) within an individual genotype.
