# Supplementary material for: Nanoscaled RIM clustering at presynaptic active zones revealed by endogenous tagging
Source: Life Sci Alliance. 2023 Sep 11;6(12):e202302021. doi: 10.26508/lsa.202302021 (PMC10494931; doi:10.26508/lsa.202302021)
Supplement: Supplementary file 5 [file LSA-2023-02021_TableS5.docx]

| **parameter** | **rim^rescue-Znf^** | **rim^HA-Znf^** | **p-value** |
| --- | --- | --- | --- |
| Brp puncta per NMJ | 382 ± 103 | 394 ± 91 | 0.665 |
| NMJ area [µm^2^] | 455 (339-551) | 507 (414-653) | 0.231 |
| n (NMJs, animals) | 24, 9 | 22, 9 |  |
| boutons per NMJ | 89 (79-115) | 72 (66-88) | 0.032 |
| n (NMJs, animals) | 23, 9 | 22, 9 |  |

**Table S5. NMJ morphology in rim^rescue-Znf^ and rim^HA-Znf^.** Presynaptic membranes were stained by α-HRP and AZs were imaged using Brp^Nc82^. Parametric data, reported as mean ± SD, and non-parametric data, reported as median (25^th^-75^th^ percentile). p-values are presented for comparison between rim^rescue-Znf^ and rim^HA-Znf^.
