# Supplementary material for: Nanoscaled RIM clustering at presynaptic active zones revealed by endogenous tagging
Source: Life Sci Alliance. 2023 Sep 11;6(12):e202302021. doi: 10.26508/lsa.202302021 (PMC10494931; doi:10.26508/lsa.202302021)
Supplement: Supplementary file 6 [file LSA-2023-02021_TableS6.docx]

| **parameter** | **rim^HA-Znf^ (ctrl)** | **rim^HA-Znf^ (phtx)** | **p-value** |
| --- | --- | --- | --- |
|  | **all AZs** | | |
| locs. per SC | 6 (4-10) | 6 (4-10) | 0.073 |
| SC area [nm^2^] | 130 (36-376) | 120 (30-361) | <0.001 |
| SC loc. density [locs./ µm^2^] | 46,582 (24,124-129,639) | 50,951 (25,000-151,971) | <0.001 |
| n (SCs, NMJs, animals) | 11,094, 18, 9 | 13,568, 19, 12 |  |
| SCs per AZ | 10 (6-16) | 11 (7-18) | <0.001 |
| locs. per AZ | 84 (51-133) | 93 (57-155) | <0.001 |
| area per AZ [nm^2^] | 3,111 (1,797-5,070) | 3,231 (1,861-5,307) | 0.070 |
| radial distance [nm] | 123 (95-167) | 126 (98-178) | 0.069 |
| n (AZs, NMJs, animals) | 893, 18, 9 | 963, 19, 12 |  |
|  |  |  |  |
| Brp locs. per AZ | 493 (287-808) | 476 (288-819) | 0.681 |
| Brp i.e., AZ area [nm^2^] | 0.092 (0.065-0.134) | 0.093 (0.064-0.138) | 0.873 |
| circularity [a.u.] | 0.66 (0.52-0.78) | 0.64 (0.49-0.76) | 0.023 |
| n (AZs, NMJs, animals) | 893, 18, 9 | 963, 19, 12 |  |

**Table S6. *d*STORM analysis of RIM^HA-Znf^ and Brp. Related to Figure 4 and Figure 5 B and C.** RIM^HA-Znf^ SCs were imaged using Alexa Fluor647 and Brp^Nc82^ clusters were marked using Alexa Fluor532. Data were derived from analysis of the whole dataset i.e., without selecting according to AZ circularity (see Material and Methods). Non-parametric data, reported as median (25^th^-75^th^ percentile). p-values are presented for comparison between RIM^HA-Znf^ ctrl and RIM^HA-Znf^ phtx groups.
