# Supplementary material for: Nanoscaled RIM clustering at presynaptic active zones revealed by endogenous tagging
Source: Life Sci Alliance. 2023 Sep 11;6(12):e202302021. doi: 10.26508/lsa.202302021 (PMC10494931; doi:10.26508/lsa.202302021)
Supplement: Supplementary file 7 [file LSA-2023-02021_TableS7.docx]

| **parameter** | **rim^HA-Znf^ (ctrl)** | **rim^HA-Znf^ (phtx)** | | **p-value** |
| --- | --- | --- | --- | --- |
|  | **AZs with circularity ≥ 0.6** | | | |
| locs. per SC | 6 (4-10) | 6 (4-9) | | 0.016 |
| SC area [nm^2^] | 139 (39-387) | 123 (32-355) | | <0.001 |
| SC loc. density [locs./ µm^2^] | 44,493 (23,753-120,319) | 49,761 (24,899-142,203) | | <0.001 |
| n (SCs, NMJs, animals) | 5,884, 18, 9 | 6,500, 19, 12 | |  |
| SCs per AZ | 8 (5-14) | 10 (6-15) | | 0.003 |
| locs. per AZ | 70 (44-114) | 81 (49-122) | | 0.019 |
| area per AZ [nm^2^] | 2,559 (1,596-4,412) | 2,706 (1,618-4,285) | | 0.614 |
| radial distance [nm] | 113 (90-140) | 108 (91-134) | | 0.455 |
| n (AZs, NMJs, animals) | 550, 18, 9 | 554, 19, 12 | |  |
|  |  |  | |  |
| Brp locs. per AZ | 437 (258-713) | 426 (262-706) | | 0.614 |
| Brp i.e., AZ area [nm^2^] | 0.084 (0.063-0.114) | 0.084 (0.059-0.115) | | 0.579 |
| circularity [a.u.] | 0.75 (0.69-0.82) | | 0.74 (0.67-0.83) | 0.394 |
| n (AZs, NMJs, animals) | 550, 18, 9 | | 554, 19, 12 |  |
|  |  | | | |
| SC c.o.m. distances [nm] | 180 (103-285) | | 169 (96-274) | <0.001 |
| n (distances, SCs, AZs) | 93,552, 5,876, 542 | | 108,618, 6,491, 545 |  |
| nearest neighbor distance [nm] | 49 (24-76) | | 44 (23-70) | <0.001 |
| n (distances, AZs) | 5,876, 542 | | 6,491, 545 |  |
| second neighbor distance [nm] | 71 (42-106) | | 66 (39-97) | <0.001 |
| n (distances, AZs) | 5,842, 525 | | 6,463, 531 |  |
| third neighbor distance [nm] | 91 (59-134) | | 84 (55-121) | <0.001 |
| n (distances, AZs) | 5,770, 501 | | 6,409, 513 |  |
| fourth neighbor distance [nm] | 107 (72-156) | | 99 (67-143) | <0.001 |
| n (distances, AZs) | 5,558, 448 | | 6,253, 474 |  |
| fifth neighbor distance [nm] | 123 (83-178) | | 116 (79-163) | <0.001 |
| n (distances, AZs) | 5,328, 402 | | 6,108, 445 |  |
| clustered SC c.o.m.s per AZ [%] | 0 (0-73) | | 50 (0-75) | 0.031 |
| n (AZs, NMJs, animals) | 542, 18, 9 | | 545, 19, 12 |  |
| SpCs per AZ | 2 (2-3) | | 2 (2-3) | 0.576 |
| n (AZs, NMJs, animals) | 247, 18, 9 | | 290, 19, 12 |  |
| SCs per SpC | 4 (3-5) | | 4 (3-5) | 0.747 |
| SC c.o.m. to SpC  c.o.m. distance [nm] | 34 (21-51) | | 32 (21-47) | 0.238 |
| n (SpCs) | 713 | | 823 |  |

**Table S7. *d*STORM analysis of RIM^HA-Znf^ and Brp^Nc82^ in AZs with high circularity. Related to Figure 5 E and Figure S3.** RIM^HA-Znf^ SCs were imaged using Alexa Fluor647 and Brp^Nc82^ clusters were marked using Alexa Fluor532. Data were derived from analysis of solely AZs with circularity ≥ 0.6 (compare Table S6 for all data). Results of supercluster (SpC) analysis in these AZs are also provided. Non-parametric data, reported as median (25^th^-75^th^ percentile). p-values are reported for comparisons between RIM^HA-Znf^ ctrl and RIM^HA-Znf^ phtx groups.
