# Supplementary material for: Nanoscaled RIM clustering at presynaptic active zones revealed by endogenous tagging
Source: Life Sci Alliance. 2023 Sep 11;6(12):e202302021. doi: 10.26508/lsa.202302021 (PMC10494931; doi:10.26508/lsa.202302021)
Supplement: Supplementary file 8 [file LSA-2023-02021_TableS8.docx]

| **parameter** | **ctrl** | | **phtx** | | **pairwise p-value** | |
| --- | --- | --- | --- | --- | --- | --- |
| **RIM^HA-Znf^, extrasynaptic SCs, all AZs** | | | | | | |
| locs. per SC | 6 (4-10) | | 6 (4-10) | | 0.992 | |
| SC area [nm^2^] | 168 (42-447) | | 163 (46-439) | | 1.000 | |
| SC loc. density [locs./µm^2^] | 38,636 (21,250-114,173) | | 38,965 (21,254-106,487) | | 1.000 | |
| n (SCs, NMJs, animals) | 8,697, 18, 9 | | 10,197, 19, 12 | |  | |
| **RIM^HA-Znf^, extrasynaptic SCs ≤ 400 nm, AZs with circularity ≥ 0.6** | | | | | | |
| locs. per SC | 7 (4-10) | | 6 (4-10) | | 0.571 | |
| SC area [nm^2^] | 168 (39-439) | | 157 (37-430) | | 1.000 | |
| SC loc. density [locs./µm^2^] | 39,059 (21,578-120,911) | | 40,524 (21,680-123,154) | | 1.000 | |
| n (SCs, NMJs, animals) | 4,675, 18, 9 | | 4,931, 19, 12 | |  | |
| **RIM^HA-Znf^, intrasynaptic SCs, all AZs** | | | | | | |
| locs. per SC | 6 (4-10) | | 6 (4-10) | | 1.000 | |
| SC area [nm^2^] | 135 (31-425) | | 125 (27-401) | | 0.007 | |
| SC loc. density [locs./µm^2^] | 46,830 (23,233-145,215) | | 51,455 (24,403-166,250) | | <0.001 | |
| n (SCs, NMJs, animals) | 11,775, 18, 9 | | 14,081, 19, 12 | |  | |
| locs. per SC | ANOVA p-value | 0.015 | | extrasynaptic vs. intrasynaptic SCs ctrl | | 0.371 |
| SC area [nm^2^] |  | <0.001 | |  |  | <0.001 |
| SC loc. density [locs./µm^2^] |  | <0.001 | |  |  | <0.001 |
|  | | | | | | |
| **parameter** | **ctrl** | | **phtx** | | **p-value** | |
| **RIM^HA-Znf^, extrasynaptic SCs ≤ 400 nm, AZs with circularity ≥ 0.6** | | | | | | |
| SCs per AZ | 7 (4-11) | | 7 (4-12) | | 0.418 | |
| locs.per AZ | 61 (31-105) | | 63 (34-105) | | 0.722 | |
| radial distance [nm] | 379 (318-447) | | 397 (332-458) | | 0.017 | |
| n (AZs, NMJs, animals) | 525, 18, 9 | | 532, 19, 12 | |  | |
| **RIM^HA-Znf^, intrasynaptic SCs, all AZs** | | | | | | |
| SCs per AZ | 11 (7-17) | | 12 (8-19) | | 0.003 | |
| locs.per AZ | 95 (62-149) | | 108 (70-170) | | <0.001 | |
| area per AZ [nm^2^] | 3,684 (2,284-5,780) | | 3,805 (2,311-6,347) | | 0.091 | |
| radial distance [nm] | 142 (108-189) | | 142 (108-196) | | 0.655 | |
| n (AZs, NMJs, animals) | 892, 18, 9 | | 961, 19, 12 | |  | |

**Table S8. *d*STORM analysis of intra- and extrasynaptic RIM^HA-Znf^. Related to Figure S2.** RIM^HA-Znf^ SCs were imaged using Alexa Fluor647 and Brp^Nc82^ clusters were marked using Alexa Fluor532. Data were derived from an alternative analysis algorithm relying on HDBSCAN for denoising of RIM^HA-Znf^ localizations (see Material and Methods, Fig. S2). Results for the population of extrasynaptic SCs > 50 and ≤ 400 nm distant from the assigned Brp^Nc82^ cluster are reported for AZs with a circularity ≥ 0.6. Non-parametric data, reported as median (25^th^-75^th^ percentile). p-values are reported for pairwise comparisons and for ANOVA on Ranks for multiple comparisons.
