# Supplementary material for: Nanoscaled RIM clustering at presynaptic active zones revealed by endogenous tagging
Source: Life Sci Alliance. 2023 Sep 11;6(12):e202302021. doi: 10.26508/lsa.202302021 (PMC10494931; doi:10.26508/lsa.202302021)
Supplement: Supplementary file 9 [file LSA-2023-02021_TableS9.docx]

| primer ID | 5‘-3‘ sequence |
| --- | --- |
| *mh_01F* | AGTTAGGTAGCGACATTTTAGAT |
| *mh_02R* | TATCTGTATTGAAATTCATAGTA |
| *mh_03F* | GTGAGTTGAGACGTTTTGTCCAA |
| *mh_04R* | GCACCAATGTGAGACAAGATATA |
| *mh_41F* | CTTCGACCGACCCGGCATCTCGTT |
| *mh_42R* | AAACAACGAGATGCCGGGTCGGTC |
| *mh_43F* | CTTCGCCTTGCGGGATACTCAGAG |
| *mh_44R* | AAACCTCTGAGTATCCCGCAAGGC |
| *mh_57F* | GTACGCTCTTCCTATTGAGTATCCCGCAAGGCAATGCCC |
| *mh_58R* | TAGAGCTCTTCTGACGCACTGGCATATAATTTATGCTGA |
| *mh_77F* | ATCTCACCTGCAAGCTCGCACTAGTCGGCGCTCGACAACAGGCAG |
| *mh_79R* | GAATCACCTGCAGAACTACGCTAGCGTCTCGCTGGCGGTGGCTCT |
| *mh_80F* | TCGCACTAGTATGCGTCATAAAGCAGCAATAAAT |
| *mh_81R* | CTACGCTAGCGAGATGCCGGGTCGGTCAGTATCT |
| *lm_28F* | ATAGTTTAGCGGCCGCGTTTGGACCCCCTTTTTCACCTCCCACTTT |
| *lm_29R* | AGGCGCGCCGAGCGGAAAGTATGGAAGCGATAGATACGA |
| *am_212F* | CTAGGTTCGAACGTACGTCGCGACTCGAGC |
| *am_213R* | CATGGCTCGAGTCGCGACGTACGTTCGAAC |

**Table S9. Primer sequences.** All primers used in this study are listed.
