# Supplementary material for: Nanoscaled RIM clustering at presynaptic active zones revealed by endogenous tagging
Source: Life Sci Alliance. 2023 Sep 11;6(12):e202302021. doi: 10.26508/lsa.202302021 (PMC10494931; doi:10.26508/lsa.202302021)
Supplement: Supplementary file 10 [file LSA-2023-02021_TableS10.docx]

| **parameter** | **RIM^HA-Znf^** | |
| --- | --- | --- |
|  | **ctrl** | **phtx** |
| SCs per AZ | 8 | 10 |
| median SC area [nm^2^] | 139 | 123 |
| SC radius [nm] | 6.7 | 6.3 |
| radial distance 10^th^ percentile [nm] | 70 | 72 |
| radial distance 25^th^ percentile [nm] | 90 | 91 |
| radial distance median [nm] | 113 | 108 |
| radial distance 75^th^ percentile [nm] | 140 | 134 |
| radial distance 90^th^ percentile [nm] | 175 | 166 |

**Table S10. Quantitative parameters derived from *d*STORM analysis of RIM^HA-Znf^ used for 2D AZ models. Related to Figure S4.**

SC area and radial distance for the same experimental datasets presented in Table S7 for AZs with circularity ≥ 0.6, extended by the SC radius that was computed under the assumption of circular SC areas as well as additional 10^th^ and 90^th^ percentiles for SC radial distances. The 2D AZ models presented in Figure S4 were based on these parameters (see Material and Methods).
